# Mean platelet volume levels in children with sleep-disordered breathing: a meta-analysis

**DOI:** 10.1186/s12887-020-02099-5

**Published:** 2020-05-11

**Authors:** Wen-Dien Chang, Chih-Hao Tseng, Yung-An Tsou

**Affiliations:** 1 Department of Sport Performance, National Taiwan University of Sport, Taichung, Taiwan; 2 Clinical Laboratory, Cheng-Ching General Hospital, Taichung, Taiwan; 3 Department of Otolaryngology-Head and Neck Surgery, China Medical University Hospital, Taichung, Taiwan; 4 Department of Audiology and Speech-Language Pathology, Asia University, No. 91, Hsueh-Shih Road, Taichung, Taiwan

**Keywords:** Pediatric sleep disordered breathing, Snoring, Mean platelet volume

## Abstract

**Background:**

Pediatric sleep-disordered breathing (SDB) correlated with respiratory conditions of snoring and hypopnea. Mean platelet volume (MPV) was an inflammatory marker, related to increased inflammatory condition of pediatric patients. Increase of MPV level may cause failure to thrive or increased upper airway infection rate. The aim of this study was to perform systematic review and meta-analysis to investigate the difference on MPV values for pediatric SDB, and compare the change on MPV after surgery in patients with pediatric SDB.

**Methods:**

A systemic review of the studies from PubMed, EMBASE, and Cochrane Library databases was conducted in March 2020, supported by reviewing of published articles for studies comparing MPV in pediatric SDB. Meta-analysis was used to compare the change of MPV in pediatric SDB, and sub-group analysis was also used to compare the MPV decrease after surgeries of adenoidectomy or adenotonsillectomy.

**Results:**

There were seven studies included in the review. Six of them including 963 subjects showed that a significant increase of MPV was noted in pediatric SDB compared to those in pediatric non-SDB (*P* < 0.05). Total standardized mean difference (SMD) in MPV between pediatric SDB and non-SDB was 0.51 (95% CI =0.30–0.72, *P* < 0.05). A significant decrease of MPV was found in pediatric SDB patients who underwent surgery (total SMD = − 0.36; 95% CI = − 0.70– -0.02, *P* < 0.05). Decreases of MPV after adenoidectomy and adenotonsillectomy were observed, but only the effect of adenotonsillectomy had a statistical significance (total SMD = − 0.72; 95% CI = − 1.18 – -0.26, *P* < 0.05).

**Conclusion:**

The MPV was significantly higher in patients with pediatric SDB, indicating the presence of increased platelet activity in pediatric SDB patients. The level of MPV could be reduced by the two surgeries, especially adenotonsillectomy.

## Background

Pediatric sleep-disordered breathing (SDB) is characterized by loud snoring and or hypopnea at night and is associated with upper airway infections [1], attention disorder [2], and failure to thrive [3]. The pediatric SDB is correlated with pediatric upper respiratory tract infection, failure to thrive, pediatric hypertension [4, 5], and attention deficiency [2]. Pediatric SDB needed to be diagnosis if pediatric patients with night time symptoms of snoring or obstructed breathing during sleep; daytime symptoms such as excessive daytime sleepiness; and associated neuropsychiatric problems as behavioral problems; agitation behavior, poor concentration, and other problems concerning to grow retardation or learning difficulties. Pediatric sleep apnea is defined as AHI > 1 per hour or hypercapnia (PaCO2 > 50 mmHg) in 25% sleep time association with snoring, paradoxical thoracoabdominal motion etc. [6]. The association of pediatric SDB caused to increase interest in its role in upper respiratory tract infection [1], and pediatric hypertension [4, 5], failure to thrive (increased catabolic status by chronic inflammation) [3], and attention deficiency disorders [2]. Platelet volume correlated with cardiovascular disease [7], and coronary heart disease [8].

Mean platelet volume (MPV) is an inexpensive and simple test, using to measure platelet size, and is a platelet activity marker. MPV was associated with the factors of cardiovascular disease risk, like diabetes [9], hypertension [10], systemic lupus erythematosus [11], and atherosclerosis [12]. MPV is also associated with increase in catabolic metabolic status [13], as well as arterial and venous thrombosis [14]. A recent study have evaluated MPV in adult patients with sleep-disordered breathing [15]. Some studies have shown a significant association between MPV and sleep-disordered breathing in adult patients [16, 17]. It is reported that adult patients with sleep-disordered breathing have higher MPV, and MPV disease after wearing continuous positive airway pressure [18], and sleep surgery by uvulopalatal flap in adult patients with sleep-disordered breathing [19]. There are very few reports that show the correlation of MVP in patients with pediatric SDB, and relationship of increase of MVP and pediatric SDB was still unclear. This meta-analysis evaluates the relationship of pediatric SDB and MPV by combining data from all the relevant articles. To the best of our knowledge, this is a first meta-analysis on this field of pediatrics. Therefore, the aim of our study was to use systematic review and meta-analysis to investigate the difference on MPV values for pediatric SDB, and compare the change on MPV after surgery in patients with pediatric SDB.

## Methods

### Search strategy and data sources

Articles about the association of pediatric SDB and MPV, were case series, retrospective cohort studies, and randomized clinical trial studies, were searched independently by two researchers. Our searching strategy was based on patient, indicator, control group and outcome (PICO): is there a difference on MPV values for pediatric SDB, and change on MPV after surgery in patients with pediatric SDB? The electronic databases of PubMed, EMBASE, and Cochrane Library were searched primarily based on the combination of the following keywords: “sleep-disordered breathing,” “adenotonsillar hypertrophy,” “Adenoid hypertrophy,” “mean platelet volume,” and “platelet” up to March 2020 and published in English. Searched literatures were limited to children from birth to 18 years. The syntaxes were adjusted according to different databases. The reference list of retrieved articles was scanned for all relevant additional articles and reviews by two researchers. The syntaxes for searches in PubMed and EMBASE were as follows:

#### PubMed

#1 (“sleep disordered breathing” [All Fields] OR “sleep-disordered breathing” [All Fields] OR “adenotonsillar hypertrophy” [All Fields] OR “Adenoid hypertrophy” [All Fields]) AND (“infant” [MeSH Terms] OR “child” [MeSH Terms] OR “adolescent” [MeSH Terms]).

#2 (“mean platelet volume” [All Fields] OR (“blood platelets” [MeSH Terms] OR (“blood” [All Fields] AND “platelets” [All Fields]) OR “blood platelets” [All Fields] OR “platelet” [All Fields])) AND (“infant” [MeSH Terms] OR “child” [MeSH Terms] OR “adolescent” [MeSH Terms]).

#3 #1 AND #2.

#### EMBASE

#1 ‘sleep disordered breathing’/exp. OR ‘sleep disordered breathing’ OR ‘sleep-disordered breathing’/exp. OR ‘sleep-disordered breathing’ OR ‘adenotonsillar hypertrophy’/exp. OR ‘adenotonsillar hypertrophy’ OR ‘adenoid hypertrophy’/exp. OR ‘adenoid hypertrophy’.

#2 ‘mean platelet volume’ OR platelet.

#3 #1 AND #2.

#4 #3 AND ([adolescent]/lim OR [child]/lim OR [embryo]/lim OR [fetus]/lim OR [infant]/lim OR [newborn]/lim OR [preschool]/lim OR [school]/lim).

### Study selection

Two researchers used search criteria to include articles of pediatric patients with sleep disordered breathing (diagnosed either by symptoms or polysomnography), and one of the main outcomes of comparison between patients with and without pediatric SDB was MPV. The second aim is to see whether adenoidectomy and adenotonsillectomy could reduce MPV level before and after surgery. Only articles published in English which survey the pediatric SDB were included. Articles that did not have MPV measure as one of the main outcomes were excluded.

### Data extraction and quality assessment

Data regarding first author, article publication year, country of study, patients population characteristics, and study setting were extracted and collected. Data were extracted by 2 researchers independently using a standardized format. Mean MPV with SD were calculated accordingly.

Subject data in the cases of pediatric SDB and control groups were extracted directly from the data provided in the included articles. As the original publication provided median value of MPV level, one researcher was directly contacted to get more information for meta-analysis. The quality of included articles were assessed using the Methodological Index for Nonrandomized Studies (MINORS) tool by two researchers independently. The MINORS was primarily used to assess the risk of bias and applicability judged as adequately reported, inadequately reported, and not reported, scoring 2, 1, and 0, respectively [20]. The highest score for noncomparative study was considered 16, and that for comparative study was considered 24. The scores of MINORS were from 9 to 20, and there was a good consensus regarding study quality.

### Statistical analysis

The comprehensive meta-analysis software (Comprehensive Meta-Analysis Version 3, Biostat Inc., Englewood, NJ) was used to perform for all statistical analyses. Descriptive statistics was used for continuous variable, and the data were presented as mean ± SD. In meta-analysis, the standardized mean difference (SMD) was used to analyze the MPV level between patients with pediatric SDB and control group as the main measure of association. Fixed or random effects models was used for pooling of the data from the included articles. The estimates of SDM were expressed with 95% confidence intervals (CIs). The heterogeneity analysis was assessed by Q statistic and I^2^ statistics, which derived from Cochran Q statistic. All analysis were two-tailed tests and *P* < 0.05 was considered statistically significant. A value of I^2^ > 50% was meant substantial magnitude of heterogeneity, and a value of P < 0.05 was meant significant heterogeneity. A sensitivity analysis was also performed to determine the impact of the quality score on the findings of this systemic review. The Egger’s test and Rosenthal’s fail-safe number were used to assess any publication bias [21, 22].

## Results

### Data search results

**Fig. 1:**
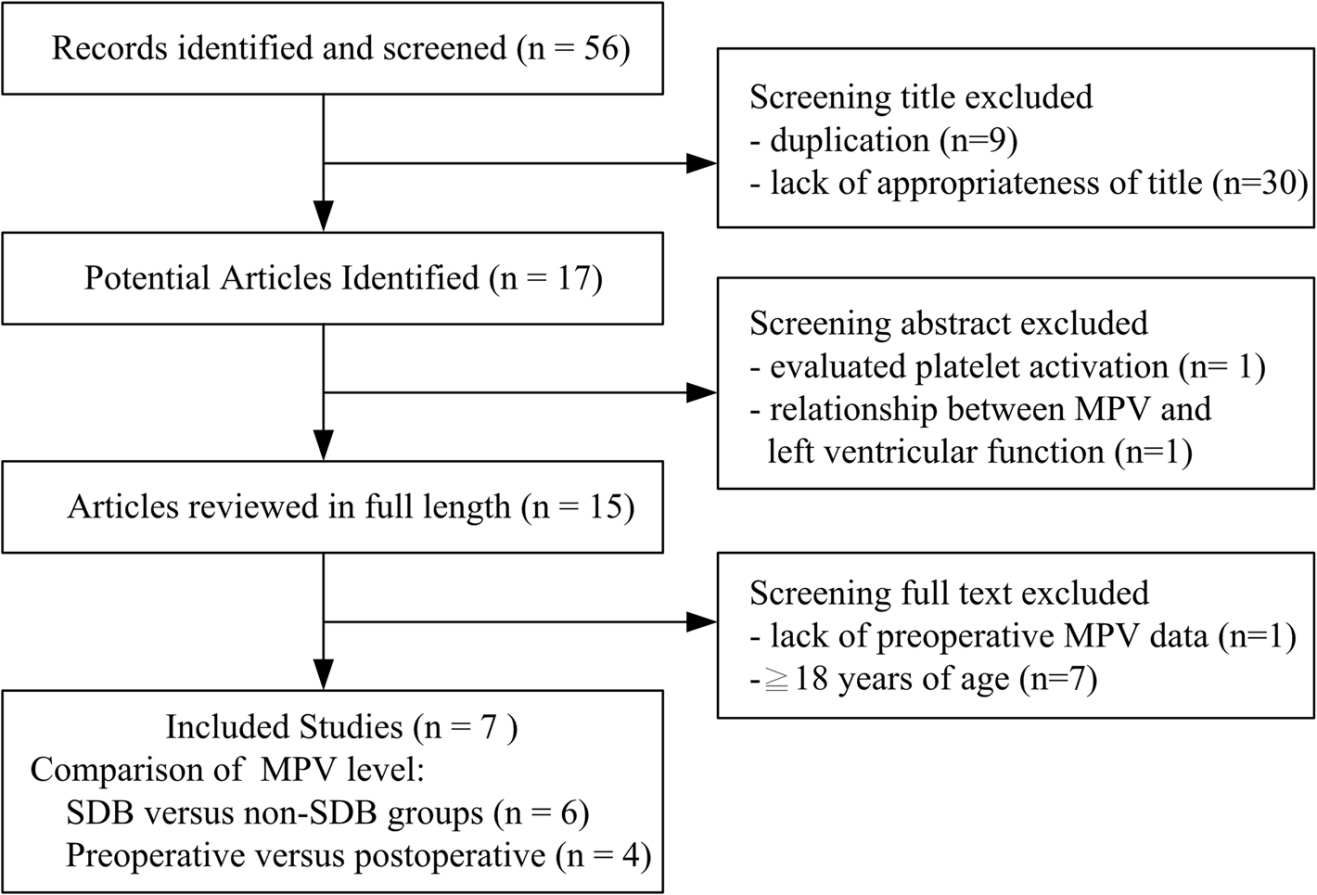
Study flow diagram: progression of papers through the review process

We used the search strategy to identify 56 articles from the electronic databases, and all articles were screened subsequently by reading titles in Fig. 1. Nine studies were discarded because of duplication, and 30 were excluded due to lack of appropriateness of title. After remaining the abstracts, 17 articles were read in detail, and another 2 articles were excluded. The excluded records were as following: evaluated platelet activation (*n* = 1), and relationship between MPV and left ventricular function (n = 1). Fifteen articles were selected in full length by 2 reviewers for appropriateness and the criteria. Finally, seven studies were reviewed [23–29].

In Fig. 1, because the study of Onder et al. was noncomparative study without data of control group [25]. Three studies focused on comparison MPV value without surgery [25, 28, 29], and four studies about MPV level change after surgery involving 744 subjects were included in the meta-analysis [23, 25–27]. Among these included trials, six studies (total subjects, *n* = 963) compared MPV value between pediatric SDB (*n* = 552) versus non-SDB groups (*n* = 411) [23, 25–29]. Four studies (total subjects, *n* = 369) compared preoperative versus postoperative values of MPV [23–26].

### Study characteristics

In Table 1, study characteristics of included articles are shown. The endoscopy was used for SDB diagnosis in four studies [23, 25–27], polysomnography was used in three studies [27–29], and cephalometry was used in one study [24]. The MPVs were measured from blood sampling within 1 h, and used EDTA to avoid EDTA-induced platelet swelling. However, the diagnosis of PDSB is only found in three studies according to apnea hypopnea index done by polysomnography [27–29]. There were five studies from Turkey [23–26, 28], one study from Italy [27], and one study from Spain [29]. All the studies published from 2014 to 2019, and study quality was evaluated using MINORS tool in Table 2.

**Table 1:** Characteristics of study design, PSDB diagnosis, and included article quality

Authors	Country	Study design	Evaluations	PSDB Diagnosis	MINORS
Kucur et al. [23]	Turkey	Prospective study/ comparative study	Endoscopy	Children had grade 3 (50 to 75% of choanal space) or 4 (75 to 100% of choanal space) adenoid hypertrophy	16
Onder et al^.^ [24]	Turkey	Prospective study/ noncomparative study	Cephalometry	Children with enlarged adenoid (adenoid/nasopharynx ratio > 0.7)	9
Soyalic et al^.^ [25]	Turkey	Prospective study/ comparative study	Endoscopy	Children had grade 3 (adenoid tissue fill 50–75% of choana) or 4 (adenoid tissue fill 75–100% of choana) adenoids, and 3+ (50–75% of airway obstruction) or 4+ (airway obstruction > 75%) tonsil hypertrophy	19
Simsek et al. [26]	Turkey	Retrospective study/ comparative study	Endoscopy	Children with adenoid hypertrophy (choanal space ≥50%) or chronic tonsillitis (repeated attacks ≥4 or 5 per year)	20
Zicari et al. [27]	Italy	Clinical study/ comparative study	Endoscopy and polysomnography	Children had primary snoring (snoring without apnea, abnormal sleep, or abnormal gas exchange) and obstructive sleep apnea (partial upper airway obstruction, intermittent complete obstruction during sleep, and loss of normal ventilation during sleep)	18
Erdim et al. [28]	Turkey	Retrospective study/ comparative study	Polysomnography	Children with predicting sleep apnea (apnea-hypopenea index > 1)	17
Barceló et al. [29]	Spain	Case-control study/ comparative study	Polysomnography	Children with snoring and obstructive sleep apnea (apnea-hypopenea index > 1, decrease of hypopnea ≥30%)	17

*PICO* patient, indicator, control group and outcome, *PSDB* pediatric sleep-disordered breathing, *MINORS* Methodological Index for Nonrandomized Studies

**Table 2:** Characteristics of subject number, MPV and study reports

Authors	Total subjects (n)	PSDB (n)	Controls (n)	MPV in PSDB	MPV in controls	Subjects with surgery (n)	MPV in post-surgery	Study reports
Kucur et al. [23]	205	105	100	8.25 ± 1.10	7.50 ± 0.90	105	7.75 ± 1.20	Significant decrease in adenoid hypertrophy *
Onder et al^.^ [24]	61	61	NA	7.5 ± 1.07	NA	61	7.70 ± 0.80	Significant decreases in white blood cell levels and upper airway obstruction scores*
Soyalic et al^.^ [25]	129	73	56	7.68 ± 1.07	7.21 ± 0.84	73	7.17 ± 0.97	Significant decreases in MPV level and platelet count*
Simsek et al. [26]	212	130	82	8.24 ± 0.94	7.76 ± 0.77	130	7.82 ± 0.93	Significant decreases in MPV, platelet distribution width, hemoglobin, and white blood cell^*,a^
Zicari et al. [27]	137	67	70	8.22 ± 0.95	7.57 ± 0.56	NA	NA	MPV levels were higher in PSDB
Erdim et al. [28]	83	45	38	7.93 ± 0.59^b^ 8.05 ± 0.68^c^	7.72 ± 0.38	NA	NA	MPV levels were higher in PSDB
Barceló et al. [29]	152	87	65	7.40 ± 0.97	7.40 ± 0.95	NA	NA	No differences in MPV

MPV expressed as mean ± SD in fL; *MPV* mean platelet volume, *PSDB* pediatric sleep breathing disorder, *NA* not available

*P < 0.05, pre- vs. post-operation in PSDB patients; ^a^ Significant decreases after adenotonsillectomy were found, but no significant decreases after adenoidectomy were noted; ^b^1 ≤ apnea-hypopenea index < 5 (*n* = 24); ^c^apnea-hypopenea index ≥5 (*n* = 21)

### Outcomes of meta-analysis

**Fig. 2:**
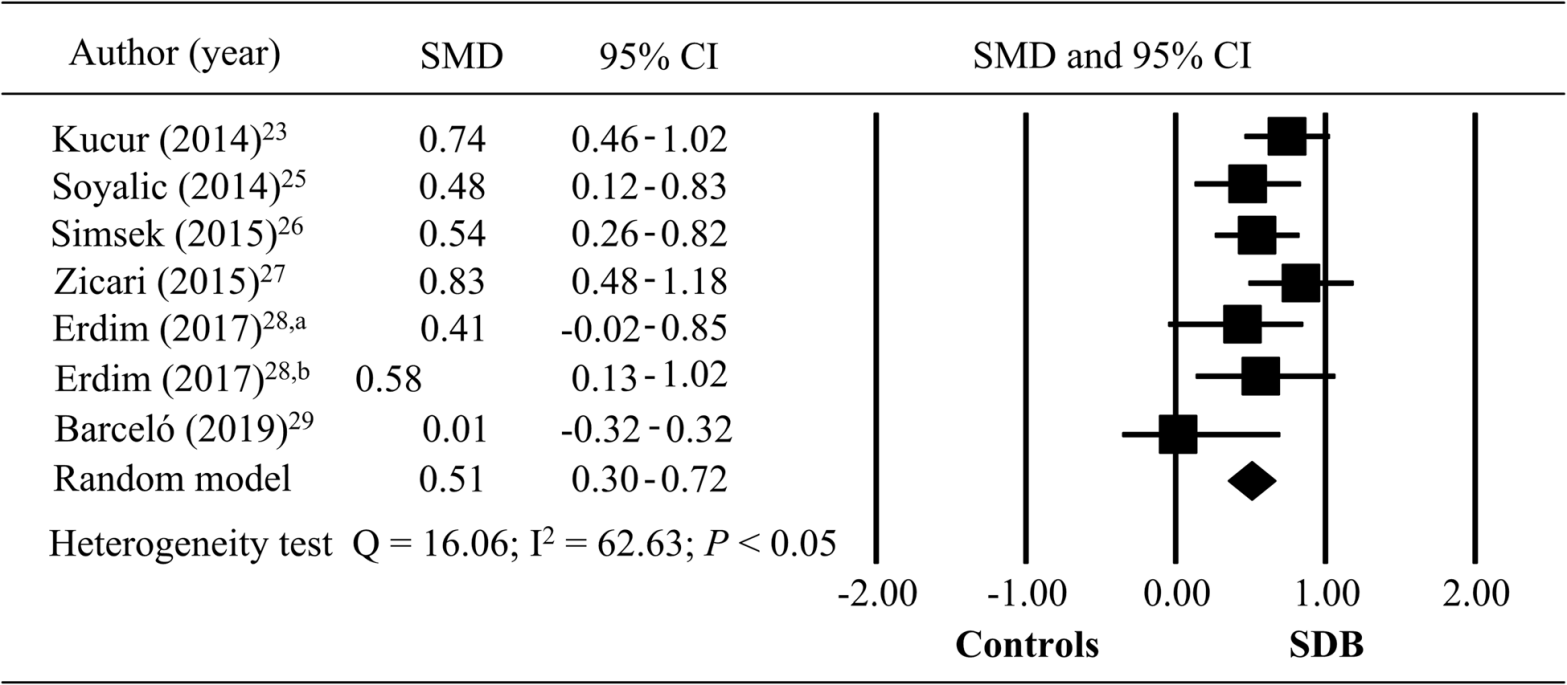
Meta-analysis of pediatric SDB and control groups (SDB, sleep-disordered breathing; SMD: standardized mean difference; CI: confidence interval)

In meta-analysis, six studies on whether the pediatric sleep surgery adenoidectomy and adenotonsillectomy could reduce the MPV level by analysis of the change in MPV level of pediatric SDB (*n* = 552) or non-SDB (controls; *n* = 411) [23, 25–29]. In the study of Erdim et al., two groups receiving adenoidectomy and adenotonsillectomy were meta-analyzed separately to compare control group [28]. In the results of Rosenthal’s fail-safe number test, the tolerance level of 35 was lower than the fail-safe number of 75, resulting publication bias did not affect meta-analysis. No significant publication bias was detected (t = 1.58, *P* = 0.25) in Egger’s regression intercept analysis. In Fig. 2, comparing patients with pediatric SDB (*n* = 552) to those with non-SDB (*n* = 449) in meta-analysis, a significantly heterogeneous in SMD of MPV values was found (*n* = 1001; Q = 16.06; I^2^ = 62.63; *P <* 0.05). The patients with pediatric SDB had significant higher MPV value than healthy control group (total SMD = 0.51; 95% CI =0.30–0.72, *P* < 0.05).

**Fig. 3:**
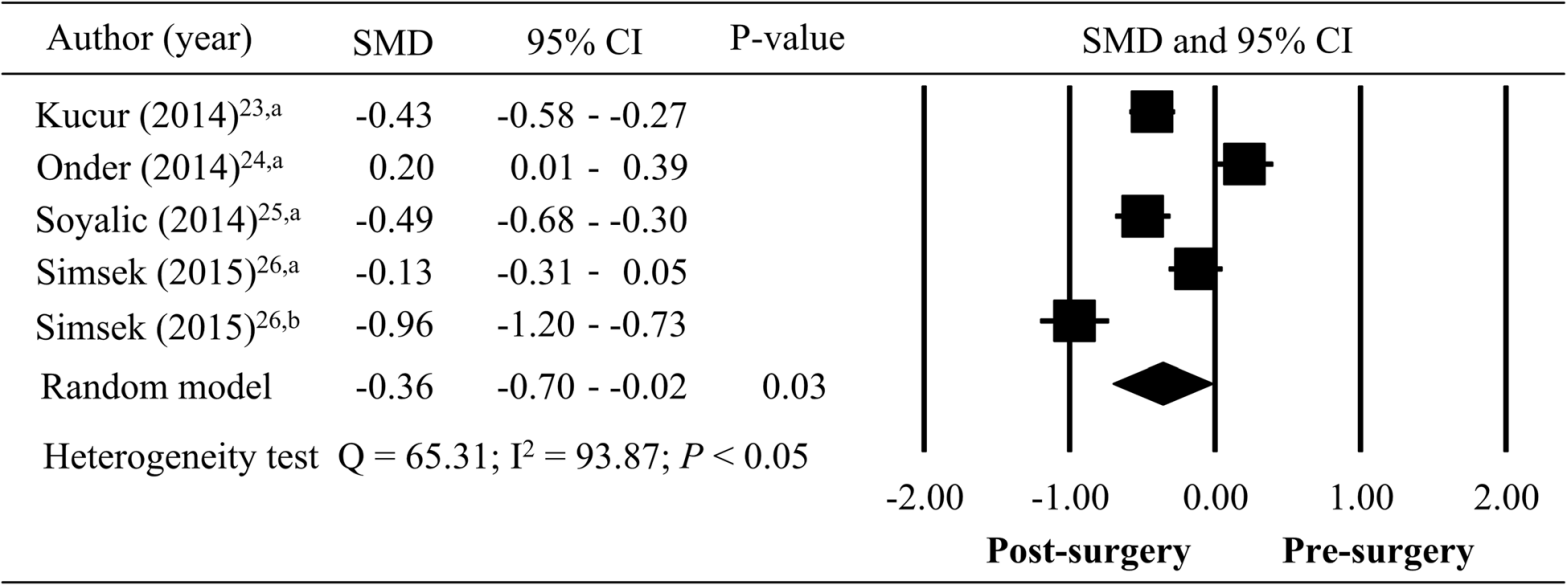
Meta-analysis of pre- and post-surgery (adenoidectomy^a^ and adenotonsillectomy^b^; SMD: standardized mean difference; CI: confidence interval)

Compared to pediatric non-SDB group, the MPV values, white blood cell levels, upper airway obstruction and adenoid hypertrophy were significantly reduced after surgery in patients with pediatric SDB among these including studies [23–26]. Of all articles, three studies were used adenoidectomy [23, 24, 26], and two studies used adenotonsillectomy for pediatric SDB [25, 26]. The MPV values were compared between pre- and post-surgery in meta-analysis (Fig. 3). Among the patients with pediatric SDB, there was significant heterogeneity in SMD of MPV value pre- and post-surgery (*n* = 369; Q = 65.31; I^2^ = 93.87; *P <* 0.05). A significant decrease of MPV found in pediatric SDB patients who underwent surgery (total SMD = − 0.36; 95% CI = -0.70– -0.02, *P* < 0.05). The results of forest plot for meta-analysis showed a significant decrease of MPV in post-surgery group.

**Fig. 4:**
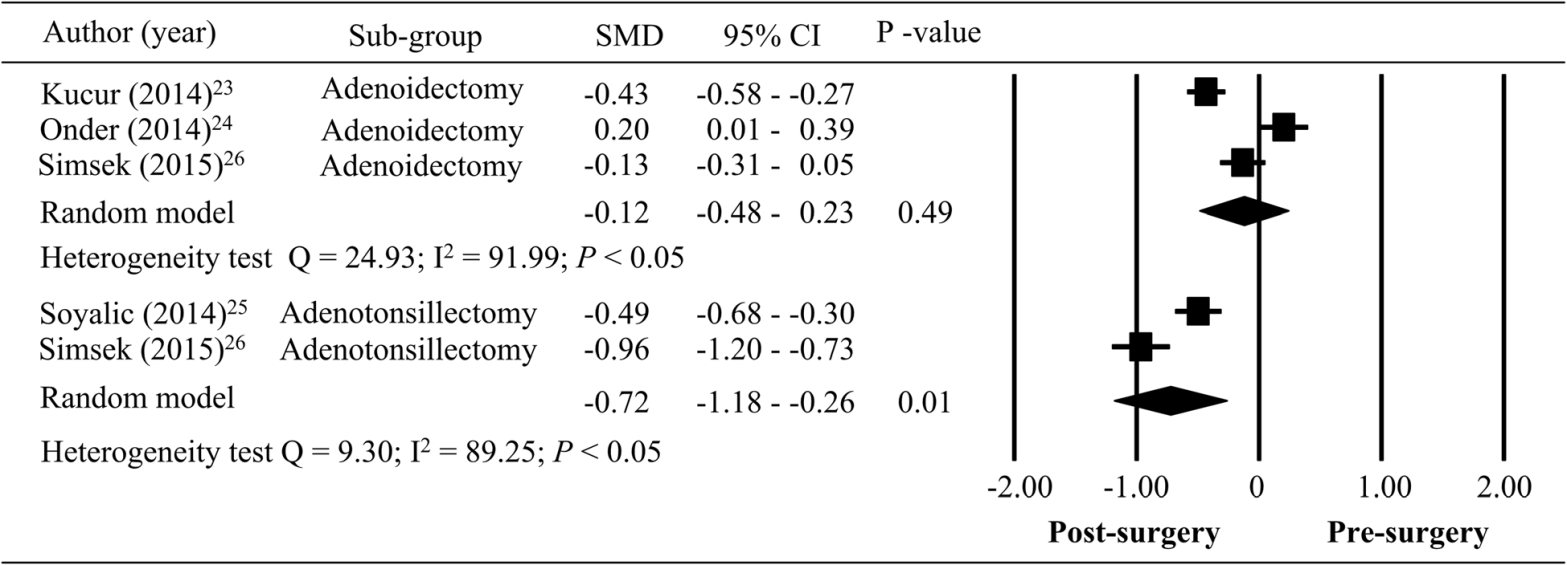
Sub-group analysis for adenoidectomy and adenotonsillectomy, pre- and post-surgery (SMD: standardized mean difference; CI: confidence interval)

In sub-group analysis, MPV values were compared before and after adenoidectomy or adenotonsillectomy. The patients received the adenoidectomy in three studies [23, 24, 26], and received the adenotonsillectomy in two studies [25, 26]. In Fig. 4, there were significant heterogeneous in SMD of MPV for the patients who underwent adenoidectomy (*n* = 235; Q = 24.93; I^2^ = 91.99; *P <* 0.05) and adenotonsillectomy (*n* = 134; Q = 9.30; I^2^ = 89.25; *P <* 0.05). Decrease of MPV after adenoidectomy was observed, but statistical significance was not reached (total SMD = − 0.12; 95% CI = − 0.48– 0.23, *P* > 0.05). However, a significant decrease of MPV were reported in the patients who underwent surgery of adenotonsillectomy (total SMD = − 0.72; 95% CI = − 1.18 – -0.26, *P* < 0.05, Fig. 4).

## Discussion

As far as we know, our study is the first systematic review and meta-analysis to the difference on MPV between SDB and healthy children, and compare the change on MPV after adenoidectomy and adenotonsillectomy in patients with pediatric SDB. Our analysis drawn from seven reviewed articles and involved 963 participants (pediatric SDB, *n* = 552; controls, *n* = 411) for meta-analysis. Compared with control group, the MPV level is significantly higher in patients with pediatric SDB in the results of meta-analysis (*P* < 0.05). The subgrouping analysis (pediatric SDB patients underwent surgery, *n* = 369), also show the adenoidectomy and adenotonsillectomy could reduce the MPV level.

MPV level is a measure of routine complete blood count, and platelets could help prevent bleeding. Of our reviewed articles, MPV level was measured as one blood parameter for pediatric SDB [23–29]. Six of them surveyed the MPV level versus control for pediatric SDB, and 4 of them surveyed the MPV level change between surgery and non-surgery groups which make this meta-analysis all the more important [23, 25–29]. Pediatric SDB attracts the sleep physicians’, pediatricians’, and otolaryngologists’ concerns much more because of its increasing prevalence rate and its sequelae if left alone which include pediatric hypertension [30], increased risk of upper respiratory tract infection [31], failure to thrive [32], and neuropsychiatric problems such as attention deficiency [33], and other mood disorders such as depression [34, 35]. Previous studies have indicated an increase in pediatric hypertension rates in patients with pediatric SDB even in non-obese pediatric groups [4, 30]. There were proatherogenic molecules, including tumor necrosis factor-a (TNF-*α*), interleukin-6 (IL-6), oxidized low density lipoprotein cholesterol, correlates to increased resistance to insulin and to active macrophage. They affected platelet volume to accelerate atherosclerosis and increase cardiovascular disease in patients with pediatric SDB and related sleep breathing disorders [36]. The pathophysiological mechanism for increased platelet volume observed in pediatric SDB is not yet clearly defined, and several explanations have been discussed in our results of meta-analysis [23, 25–29]. Pediatric SDB is related to chronic inflammatory condition such as chronic tonsillitis and chronic adenoiditis, with raised in various inflammatory mediators, such as TNF-*α,* IL-1 and IL-6 [36]. Platelets have been reported to activate these inflammatory cytokines and chemokines by changing in size, measured as MPV [36].

Increased MPV is also associated with poor outcomes after a stroke and a higher risk of coronary stent restenosis rate, which is related to vessels’ endothelial dysfunction. We did not see these associations in patients with pediatric SDB; however, we did find the association of pediatric vessels endothelial dysfunction in SDB children, which might lead to cardiovascular diseases [37, 38]. There are possible inflammatory cytokines such as renin, angiotensin, VEGF, insulin growth factor, TNF-alpha triggered by increased platelet volume rendered change blood pressure in patients with pediatric SDB. A related study also showed a positive correlation between activated platelet function to elevated MPV [38].

In our meta-analysis, MPV level is significantly higher in pediatric SDB patients compared to control group, revealing the presence of increased platelet activity in pediatric SDB patients. However, MPV level was measured as a clinical blood marker for elevated blood pressure, and it is needed to establish the pediatric hypertension in patients with pediatric SDB. There were insufficient information to infer conclusion on increased MPV in patients with pediatric SDB was responsible for higher blood pressure events in meta-analysis from six articles [23, 25–29]. Further studies are required to answer and discuss this. We also found the adenotonsillectomy significant reduced MPV when compared to adenoidectomy alone for patients with pediatric SDB. We presumed that this might due to more patency of upper airway and decreasing the hypoxic condition that leads to deactivation of platelet function. The other possible explanation is that eradicating inflammatory tissues (adenoid plus palatine tonsils) and the resulting decrease in residual inflammatory tissue ceases the active activation of platelet function and leads to decreased MPV.

**Fig. 5:**
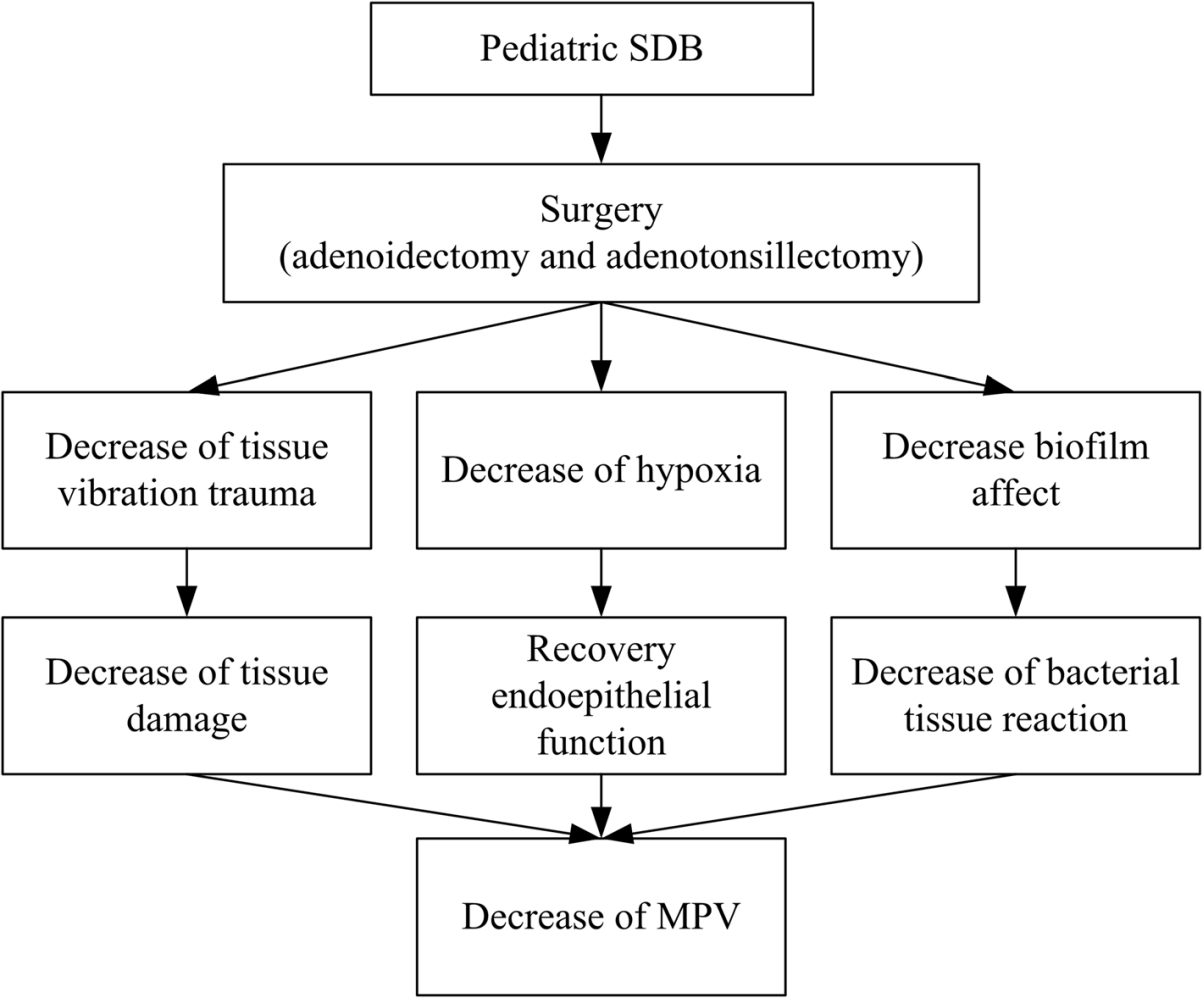
The proposed reasons of adenoidectomy and adenotonsillectomy influencing MPV

In our review, we found chronic tonsillitis and adenoiditis in pediatric patients is highly correlated to pediatric sleep disordered breathing. The children with SDB were often to have adenoid or tonsillar hypertrophy, which could be diagnosed endoscopically, and most of them suffered from snoring, obstructive sleep apnea, or a chronic inflammatory condition such as chronic tonsillitis or chronic adenoiditis. Surgical adenoidectomy or adenotonsillectomy could decrease tissue vibration trauma, decrease hypoxia, and even eradicate hidden infection sources such as biofilm. These could subsequently decrease tissue vibration damage, help patients recover endothelial function and even decrease bacterial tissue reactions. All of these factors contribute to decrease the level of MPV (Fig. 5). This will help reduce the pediatric inflammatory condition and improve oxygenation during night time sleeping and decrease the subsequent upper airway infections.

### Limitations

In our review, we found that there is a broad-spectrum MPV level, ranging 6.0 ~ 9.36 fL. An essential limitation was the effect of severity of pediatric SDB on the value of MPV in patients with pediatric SDB, as not all of the included studies proved pediatric SDB by polysomnography or sleep questionnaire. In addition, most of papers identified for meta-analysis were from Turkey and one from Italy and two were the same group authors, this might cause selection bias but there were few studies focus on MPV relating to the treatment outcomes and severity of pediatric SDB, therefore we could only use these studies for meta-analysis. By the way, we also did a subgroup analysis on whether surgery affected the MPV level when compared with no surgery. Although the MPV was still higher in patients who underwent pediatric SDB surgery, the MPV level decreased significantly after surgery. The effects of inflammatory markers on MPV were also not analyzed in out study. Another limitation in this review is that measured MPV values were so various from different laboratories that SMD was used for standardizing in our meta-analysis.

## Conclusions

This study suggests that the MPV was significantly higher in patients with pediatric SDB, indicating the presence of increased platelet activity in pediatric SDB patients. The level of MPV could be reduced by the two surgeries, especially adenotonsillectomy. The increased MPV is associated with increased blood pressure and inflammatory markers in pediatric SDB patients were needed to be studied in the future.

## Data Availability

All data generated or analyzed during this study are included in this published article.

## Abbreviations

PICO: Patient, indicator, control group and outcome
SDB: Sleep-disordered breathing
MINORS: Methodological Index for Nonrandomized Studies
MPV: Mean platelet volume
SMD: Standardized mean difference
CI: Confidence interval
IL-6: Interleukin-6
TNF-*α*: Tumor necrosis factor-*α*
